# Comparison of postoperative morbidity between piezoelectric surgery and conventional rotary instruments in mandibular third molar surgery: a split-mouth clinical study

**DOI:** 10.4317/medoral.24085

**Published:** 2021-03-27

**Authors:** Yakup Gulnahar, Aysan Lektemur Alpan

**Affiliations:** 1 DDS, PhD. Department of Maxillofacial Surgery, Faculty of Dentistry, Erzincan Binali Yıldırım University, Erzincan, Turkey; 2 DDS, PhD. Department of Periodontology, Faculty of Dentistry, Pamukkale University, Denizli, Turkey

## Abstract

**Background:**

The extraction of impacted third molar teeth is a common procedure in maxillofacial surgery. The aim of this study was to compare of piezoelectric surgical technique with the one with conventional rotary instruments in terms of edema, trismus and pain, in mandibular third molar surgery.

**Material and Methods:**

20 individuals with symmetrically impacted lower mandibular third molars and 40 teeth were included in the study. Third molars on the left side of each patient were removed with piezosurgery, while the counterparts on the right side were removed with conventional rotary instruments. Postoperatively, the same antibiotic, analgesic, and mouthwash were recommended to both groups. Ultrasound, edema, trismus measurements were performed before surgery, postoperative, postoperative day 2 and postoperative day 7. VAS scale was used to evaluate the pain.

**Results:**

The average age of 20 individuals included in the study was found to be 21.85 ± 3.08 years. The operation time of the individuals who underwent the surgery with conventional rotary instruments was found to be 12 minutes 31.70 ± 167.03 seconds, and the operation time in the Piezosurgery group was 19 minutes 10.60 ± 306.59 seconds. There was no significant difference between the two groups in terms of trismus, edema, and pain.

**Conclusions:**

Piezosurgery is a safe method that can be used in molar removal, but in this split-mouth study, it is not found advantageous in terms of postoperative morbidity due to the longer working time compared to the one performed with conventional rotary instruments.

** Key words:**Edema, impacted third molar, pain, piezosurgery, trismus.

## Introduction

Full or partially impacted teeth in the mouth cause various complications such as pericoronitis, caries, periodontal problems of adjacent molar, germination disorders and orthodontic problems (1). Therefore, third molar surgery is the most applied procedure in maxillofacial surgery. Main complications regarding third molar dental surgery are trismus, edema and pain. It is argued that the factors that combine these complications are complex, but the main factor is due to the inflammatory process caused by surgical trauma (2).

Various researchers have carried out various studies to reduce postoperative morbidity. These studies are related to; preoperative use of antibiotics, various flap techniques, using conventional rotary instruments with high or low speed, performing drain application to the operation site or not (3), postoperative use of ice (4), using systemic or topical cortisone (5,6) and using Er -YAG lasers (7).

In maxillofacial surgery, bone incisions are made with mechanical tools such as saws and burs. Mechanical tools have harmful effects due to the high amount of heat generated during the bone incision. This temperature may eventually cause marginal osteonecrosis, as it ultimately inhibits bone regeneration (8). Therefore, in the past, serious experimental efforts have been made to develop better osteotomie devices in response to the need for more precise and safe osteotomie in bone surgery (9).

In the field of dentistry, ultrasonic devices found use mainly in periodontology and endodontics after the cutting effects of high-frequency sound waves on dental hard tissues were observed in 1953. Piezosurgery is a technique that enables safe and effective osteotomies using ultrasonic vibrations. Due to its micrometric and selective cutting, the piezosurgery unit provides a safe and sensitive osteotomy without causing osteonecrotic damage. The device works only on mineralized tissues while protecting soft tissue and maintaining blood supply (9,10).

Minimally invasive surgery is essential for reducing tissue trauma and patient morbidity. In recent years, with the inclination of modern medicine towards minimally invasive surgery, the use of ultrasonic waves for bone insicions has gained importance in oral and maxillofacial surgery, on account of that ultrasonic micro-movements do not show any visible damage to adjacent soft tissues (11).

Working with ultrasound frequency, this device cuts hard tissue with micro-vibrations between 60 and 200 mm/s and in the frequency range of 24 to 29 kHz; and it solely targets mineralized tissues without damaging nerves, vessels and soft tissues (12). The tissue-selective and less heat-generating structure of piezosurgery cause less bleeding. It is advantageous to use piezosurgery in plastic surgery procedures such as genioplasty or rhinoplasty with high aesthetic expectations (13). However, its main disadvantages include high cost and long operating time (14). In addition, in a histopathological study, it was found that piezosurgery caused less edema but more tissue necrosis than the conventional method (15). Based on the known advantages of piezosurgery, we therefore hypothesize that pieosurgery would reduce postoperative morbidity in terms of pain, edema, and trismus in mandibular third molar surgery. In this study, we aimed to determine the effects of piezoelectric and conventional surgical techniques on postoperative morbidity.

Materıal and Methods

Permission was obtained from the Ethics Committee of Cumhuriyet University Faculty of Dentistry (No. 26 dated 18/11/2008) for the study protocol. Our study was planned as a prospective split-mouth study. All procedures were performed in conformity with the rules of the Declaration of Helsinki. This study was performed on patients who applied to Sivas Cumhuriyet University Faculty of Dentistry, Department of Oral and Maxillofacial Diseases for the removal of impacted mandibular third molars. Only those who were over 18 and had no systemic disease were included in the study. The patients had similar type and class of impaction on both sides of mandible and the uniform thickness of the bone that must be removed seen in the panoramic image according the Pell and Gregory classification (16). Patients who were smoking, pregnant; who had chronic diseases or severe periodontitis; and who were using analgesics or anti-inflammatory were excluded. All operations were carried out by the same physician under the same surgical and sterilization disciplines, under equal operating conditions. A written informed consent was obtained from all of the patients.

Based on the reference article, a minimum of 18 people were included in each group, with 1.26 effect size and 95% power (17). In the study, 20 patients underwent an operation for the removal of 40 impacted mandibular third molars which were symmetrically impacted in both right and left jaws. The symmetries of the teeth were evaluated according to the radiographic views of the anatomical positions of the third molar (18). During the operations, impacted mandibular third molars on one side were extracted using conventional surgical methods while the rest on the other side were extracted with a piezosurgery unit (Esacrom, Bologna; Italy). Ascertaining random instrument sequence, coin toss method used for randomization. The period from taking the first patient to the recovery of the last patient is 6 months. At least four weeks of recovery period was expected in the same patient for the removal of the second tooth.

- Surgery Procedure

The patients were asked to rinse the oral cavity with a 2% povidone-iodine solution 5 minutes before the procedure. During the operations, a local anesthetic agent containing 40 mg / ml Articaine HCl and 0.012 mg / ml epinephrine HCl (Ultraca DS forte-Aventis Pharmaceutical Tic. Inc., Istanbul, Turkey) was used as a local anesthetic solution in all groups. After the anesthesia of the n. alveolaris inferior and buccal nerve was achieved, the buccal flap was lifted with the “L” incision, the bone was removed with the help of a steel rod and fissure bur and the molar was extracted in conventional surgery group. In the piezosurgery group, piezosurgery (EMS, Piezon Master Surgery, Switzerland) unit installed with ES001 and ES005 inserts and the molar was extracted. The flap was closed primarily with a 3-0 silk suture. The time between the first incision and the last suture was recorded as the operation time. For infection control, when necessary, considering patients with penicillin allergy and stomach problems, and in order to ensure the standardization, clindamycin HCl 150 mg (from Kline, Science, Istanbul, Turkey) 4x1, paracetamol 500mg (minoset, Bayer, Turkey) 3x1 and chlorhexidine gluconate% 0.2 (Klorhex, Drogs, Ankara, Turkey) 2x1 were prescribed. The physical therapy method (cold therapy) was not recommended to any patient included in the study.

- Postoperative Edema

Postoperative edema was measured (cm) the linear distances from the angle of the mandible to the lateral canthus of the eye and from the tragus to the corner of the mouth and from the tragus to the pogonion (19). Postoperative edema was also evaluated with ultrasonography which is noninvasive, painless, rapid and inexpensive technique without any known deleterious biological effects (20,21). In order to evaluate the edema with ultrasound device, preoperative and postoperative soft tissue measurements were recorded at Sivas Cumhuriyet University Faculty of Medicine, Department of Radiology with the same device, by the same physician and with minimal pressure on the skin. For this, GE Logiq 9 (USA) brand, ultrasonography, and Sony (Japan) brand printer (UP-D897) devices were used. In each patient, the distance between the skin surface and the bone surface was measured from the fixed point marked on the skin in the sitting position and the patient's jaw muscles were not contracted, using the 10 MHz probe before and after the operation on the 2nd and 7th day. While determining the fixed point, the probe was placed 15 mm in front of the angulus mandible, which is parallel to the lower edge of the mandible and placed 15 mm in front of the angulus mandible, was taken as reference. In other words, the 30-35 mm front side of the angulus mandible was determined as the reference point. To increase the contact between the probe and the skin, the gel was applied to the masseter muscle area to be measured.

- Evaluation of Trismus

Caliper (VIS-Poland) was used to measure the trismus degree. The evaluation of the postoperative trismus, as it was done to check edema, was performed preoperatively and also on the 2nd and 7th postoperative days by the method of measuring mouth opening. The patients were asked to open their mouths as much as possible, and the distance between the incisal edges of the upper and lower central incisors was measured in millimeters with the help of a caliper. This process was repeated 3 times for each measurement and the arithmetic mean of the values found was taken.

- Postoperative Pain

A visual analog scale (VAS) of 100 mm was used to evaluate postoperative pain. 0 means "no pain" in this scale while 100 equals the worst pain imaginable (22). The patients were asked to mark the amount of pain at this scale on the 30th minute, 3rd, 6th, 9th and 24th hours following the operation and also on the 2nd, 3rd,4th,5th,6th and 7th days. At the end of the operation, patients were given forms that gave information about postoperative care. In another form, the patients were asked to rate the severity of pain at the specified hours.

- Statistical analysis

SPSS v.21.0 (SPSS Inc., Chicago, IL) was used to analyze the data. Data normality was tested using the Shapiro-Wilk test. As VAS measurements are nonparametric, related samples Wilcoxon test was used for measurements of the same group. Repeated measurements ANOVA used for trismus and edema measurements. For paired comparisons, the Mann–Whitney U test for nonparametric measurements and independent sample t test for parametric measurements were used. Statistical significance was established at p < 0.05.

## Results

20 individuals with 40 symmetrically impacted mandibular third molars were included in the study. All patients were observed during the study. The average age of 20 individuals included in the study was 21.85 ± 3.08 years. 7 (35%) of these individuals were male and 13 (65%) were female. The conventional method was used for 8 (40%) right and 12 (60%) left mandibular third molars whereas the piezosurgery method was used for 12 (60%) right and 8 (40%) left mandibular third molars.

- Operation Durations

The average operation time was 12 minutes in the conventional group and the average operation time was 19 minutes in the piezosurgery group. There was a significant difference between these two methods in terms of operation time (*p* = 0.001, *p* <0.05).

- Evaluation of Trismus

**Figure 1:**
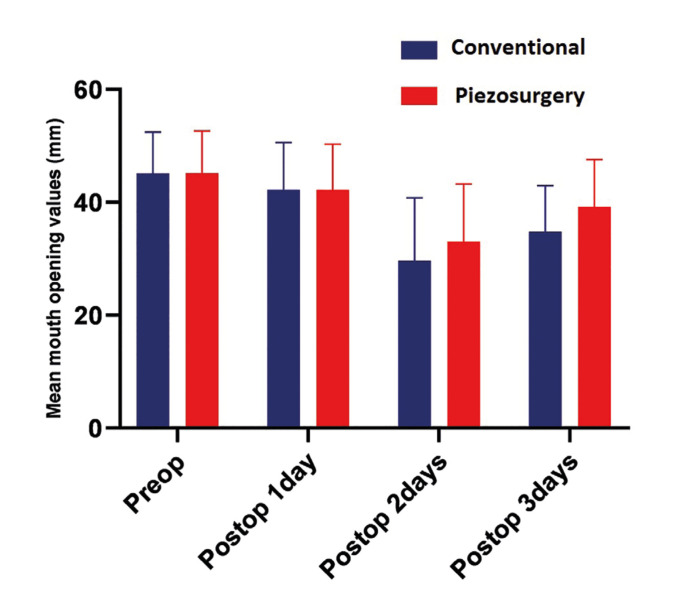
Mean mouth opening values of groups.

In the postoperative 1st day measurement, 6.38% and 6.46% reduction in mouth opening were observed in the conventional and piezosurgery groups, respectively. In the postoperative 2nd day measurements, there was a 34.18% and 26.92% reduction in mouth opening compared to the initial measurements. On the 7th postoperative day, this reduction was reduced to 22.1% and 13.1%. When all time periods are considered, the difference between the two groups was not statistically significant (*p*> 0.05) (Fig. 1).

- Evaluation of Edema

According to the average of the measurements of Tragus-Mouth Corner, Tragus-Jaw Tip, Angulus-Eye Corner, which we made for the measurement of edema, on preoperative, postoperative, postoperative 2nd day and 7th day; an increase of 0.25% in the postoperative measurement, 2.18% in the postoperative 2nd day measurement, and 0.01% in the postoperative 7th day measurement were observed in the conventional group. In the piezo group, an increase of 0.08% was observed in the postoperative measurement, 0.93% in the postoperative 2nd day measurement and 0.16% in the postoperative 7th day (Table 1).

**Table 1:**
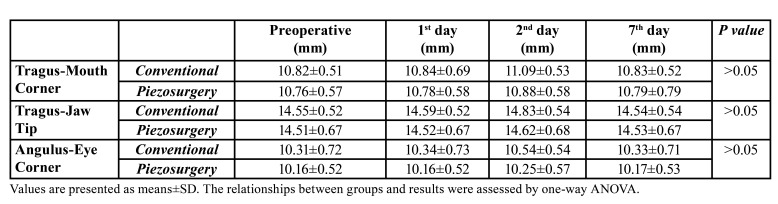
Result of edema measurements.

- Evaluation of Edema with Ultrasound

**Figure 2:**
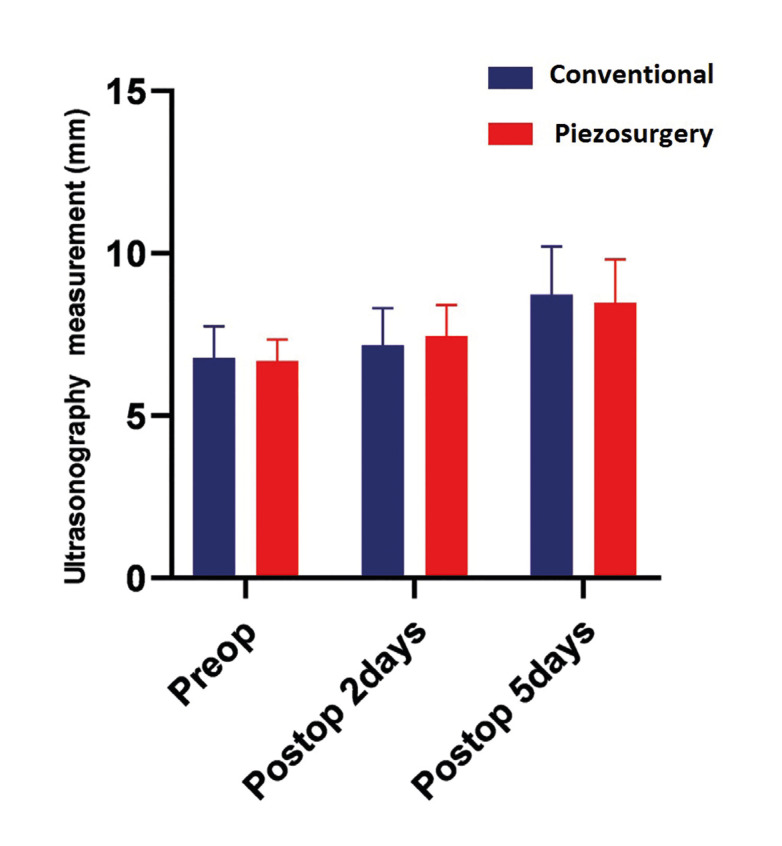
Ultrasonography data of groups.

In order to evaluate the edema, ultrasound measurements of the masseter muscle and subcutaneous were performed on preoperative, postoperative and postoperative 2nd day. When compared to the preoperative measurements (6.15 mm) by taking the average of the two values; In group A, an increase of 16.5% was observed in the postoperative measurement (7.17mm) and a 46.8% increase in the postoperative 2nd day measurement [9.03]. In group B, an increase of 11.5% was observed in the postoperative measurement (7.45 mm) compared to the preoperative measurement (6.68 mm), and an increase of 26.8% in the postoperative second day measurement (8.47 mm) (Fig. 2).

- Evaluation of Pain

**Figure 3:**
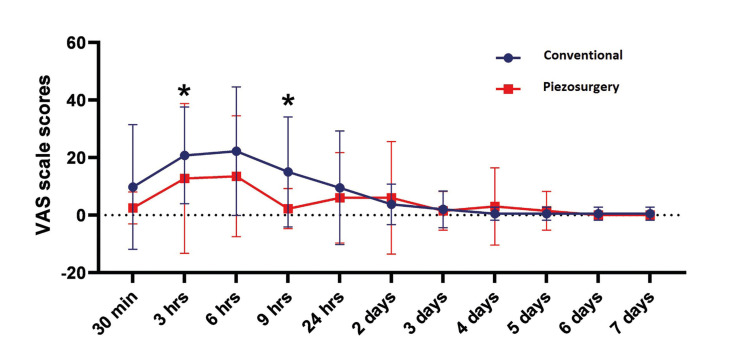
Mean VAS values of groups. *p<0.05 between two groups.

Considering the findings related to pain, there was a significant difference between the two groups at 3rd hour and 9th hour (*p* <0.05). In the data up to 24 hours, the conventional method created more pain than piezosurgery, but this curve continued at almost the same level after 24 hours. The pain level reached a peak in 6 hours in both groups and continued to decrease after that (Fig. 3. In the data of the first 24 hours, the conventional method created more pain than the piezosurgery method, but this curve continued at almost the same after 24 hours. The pain reached its peak level in the first 6 hours in both groups and continued to decrease after that (Fig. 3).

## Discussion

In maxillofacial surgery, the extraction of mandibular third molars is often performed. While conventional rotary handpieces are used for these surgeries, the use of piezosurgery has gained popularity with its selective cutting feature. In this split-mouth study, two surgical methods were compared with their advantages and disadvantages in terms of complications after mandibular third molars extraction, and according to the results of the study, no significant difference was found between the two methods in terms of postoperative edema and trismus. When the postoperative pain was evaluated, less pain was observed in the piezosurgery group at the 3rd and 9th hours between the two groups.

Piezosurgery has many advantages mentioned in the literature. First of all, it does not produce excessive heat during the operation; secondly, continuous irrigation makes the operation area more visible, thirdly, it does not damage the soft tissues, especially inferior alveolar nerve, periosteum, Schneiderian membrane, and oral mucosa, which are close to the surgery area, and it reduces bleeding in the surgical area. Many studies are investigating postoperative morbidity after the removal of mandibular third molars. Pain, swelling, and trismus were generally examined in these studies. In a study by Goyal *et al*. (23), it was reported that the use of piezosurgery significantly reduced postoperative pain, swelling, and trismus. In a similar study, it was revealed that piezosurgery caused an increase in the quality of life of patients and decreased postoperative pain, trismus, and swelling (24). In a split-mouth study, Menziletoglu *et al*. (25) investigated the effect of piezosurgery on postoperative pain, swelling, trismus and the comfort of the patient, and according to the results, piezosurgery did not provide a superiority over conventional method.41 In our study, only 3 hrs and 9 hrs VAS results was significantly lower in the favor of piezosurgery but all other investigated data in terms of postoperative pain, trismus and edema, differences were insignificant. While other evaluations, other than VAS, are subjective, VAS is a personal and objective evaluation method. Therefore, different results can be found in studies involving VAS. Similar to our study results, Taskın *et al*. (26) did not find a statistically significant difference between the two groups in terms of postoperative edema in a cohort of 90 patients (45 conventional and 45 piezosurgery). Bhati *et al*. (27) compared piezosurgery and conventional methods in 30 patients; parameters such as mouth opening (interincisal opening), pain (visual analog scale VAS score), swelling, the incidence of dry socket, paresthesia and duration of surgery were examined and piezosurgery showed a significant difference only in terms of VAS. The prolongation of the operation time compared to the conventional method is among the most important disadvantages of piezosurgery (14). In our study, the piezosurgery time was found to be significantly higher than the conventional method. Longer exposure of the bone may limit the advantages of this technique. Additionally, performing osteotomy on mandibular third molars and entering that narrow area may take longer and extend the duration of the operation due to the design of the piezosurgery inserts. Longer surgery causes more manipulation of the tissues and, consequently, results more inflammatory responses (28). The duration of surgery is predictive not only for the amount of edema but also for pain and trismus intensity. This is due to intraoperative complications directly related to a larger trauma or an increase in the duration of surgery (28).

Some authors claim that facial surgery in young individuals results in less difficulty in the procedure and, consequently, less surgical trauma and less edema. Elderly patients have a prolonged inflammatory process, and therefore there is a slower reduction in edema (29). In our study, the mean age of the patients was 21.85 ± 3.08 years and they were quite young, and the absence of a significant difference in edema and trismus may be due to these individuals' immune systems being better than the elderly and faster resolution of the inflammatory process. Surely postoperative edema and complications are not linked to only one point. The surgeon's ability to use that instrument efficiently also gains importance in this regard. Since piezosurgery units are more expensive than conventional rotary instruments, not every surgeon may have used this unit sufficiently. Furthermore, since all training are given primarily with conventional rotary instruments, the surgeon's experience may be more in that regard and may cause a decrease in postoperative complications even though there is an indirect effect (30).

Piezosurgery is a safe alternative method that can be used for the removal of impacted mandibular third molars. Despite this fact, postoperative pain did not provide a significant benefit in terms of edema and trismus. The prolonged surgical process, the price of these units and the need for intensive surgical experience are the disadvantages of piezosurgery. However, thanks to its selective cutting feature, piezosurgery can be preferred if there is a third molar near the anatomical formations.
